# Enhancing fluconazole reactivation against *Candida auris*: efficacy of *Cinnamomum zeylanicum* essential oil versus cinnamaldehyde

**DOI:** 10.1128/spectrum.00176-24

**Published:** 2024-03-14

**Authors:** M. Di Vito, R. Rosato, S. Rizzo, M. Cacaci, A. Urbani, M. Sanguinettii, F. Bugli

**Affiliations:** 1 Dipartimento di Scienze Biotecnologiche di Base, Cliniche Intensivologiche e Perioperatorie, Università Cattolica del Sacro Cuore, Rome, Italy; 2 UOC Chimica, Biochimica e Biologia Molecolare Clinica, Dipartimento di Scienze Biotecnologiche di Base, Cliniche Intensivologiche e Perioperatorie, Fondazione Policlinico Universitario A. Gemelli IRCCS, Rome, Italy; 3 Dipartimento di Scienze di Laboratorio e Infettivologiche, Fondazione Policlinico Universitario A. Gemelli IRCCS, Rome, Italy; Università Roma Tre, Rome, Italy

**Keywords:** antifungal resistance, azole, natural compound

## LETTER

*Candida auris* is a species of yeast belonging to the larger *Candida* genus. This yeast, in addition to persisting in environments and on patients for prolonged periods, has a high inter-patient transmissibility and a high invasive potential. Furthermore, the ability to form biofilm together with thermotolerance, osmotolerance, and filamentation characteristics outlines its high virulence (1). Similar to other yeasts, it assumes the role of an opportunistic pathogen, posing a threat to the well-being of immunocompromised patients within hospital settings. In fact, it is known that candidiasis is the most frequent cause of fungal infections in intensive care units. In immunocompromised patients, *C. auris* infection can quickly become serious with high morbidity and mortality (2). In the world, the rapid spread of *C. auris* is closely linked to its resistance to antifungal drugs such as fluconazole (90% of isolates), amphotericin B (30% of isolates), and echinocandins (5% of isolates) (3). Currently, the implementation of solid prevention and control practices is the most pursued strategy to avoid running into a lack of therapeutic resources caused by growing resistance to antifungals (1).

In this context, a comprehensive exploration of the biology of *C. auris* and the pursuit of novel therapeutic approaches are imperative to mitigate its rapid spread. Recent scientific evidence points to the environmental origin of this species, suggesting that its rapid dissemination may have been facilitated by climate change, as it appears to have adapted and become more resistant to elevated temperatures. This thermotolerant characteristic could underlie the yeast’s ability to overcome the mammals’ thermal barrier adopted to protect themselves from the colonization of environmental fungi. Furthermore, literature data highlight the importance of the skin microbiota balance to counteract *C. auris* spread. In fact, unlike other *Candida* species, this yeast is skilled in colonizing the skin which becomes the reservoir for diffusion in environments and between patients (4).

Considering the growing development of resistance, the great challenge is to combine new scientific acquisitions to clarify both the colonization and the resistance mechanism to develop more efficient drugs. The few therapeutic resources against *C. auris* push researchers toward the development of synergistic drug combinations to find possible alternatives in clinical therapies (5). Our group has recently demonstrated that the resistance to fluconazole can be overcome by combining small doses of *Cinnamomum zeylanicum* essential oil (CZ-EO) obtained from bark or its major active ingredient (cinnamaldehyde = CIN), with therapeutic doses of the drug (Table 1) (6). Comparable results were obtained by testing the activity of a fraction obtained from the CZ-EO (FR2) characterized by only three active compounds: CIN, o-methoxycinnamaldehyde (OM) and cinnamyl acetate (CA). Data obtained by checkerboard tests (Table 1) developed to better understand the role of each active compound in the synergy with fluconazole were reported in this letter. CIN showed antifungal activity when tested against the 10 strains of *C. auris* used in the previously published article. Otherwise, the other components showed no antimicrobial activity (data not shown). Previously published data showed that both CZ-EO and FR2 were synergistic with fluconazole, while CIN was only additive. New data showed that by mixing OM or CA to CIN, in the same concentrations present in FR2, the FIC values remain additive. Whereas by mixing the three active ingredients, the value becomes synergistic (<0.5) confirming the previous value obtained by studying FR2 activity.

**TABLE 1:** Checkerboard titration test between natural compounds and fluconazole^*a*^

		MIC		Combination		FIC		FICI
	AF	C (% vol/vol)	AF (μg/mL)	C (% vol/vol)	AF (μg/mL)	C (% vol/vol)	AF(μg/mL)	
CZ-EO	Fluco	0.07 ± 0.03	n.c.	0.02 ± 0.01	0.45 ± 0.32	0.26 ± 0.14	n.c.	n.c
FR2	Fluco	0.03 ± 0.01	n.c.	0.01 ± 0.00	0.72 ± 0.67	0.17 ± 0.14	n.c.	n.c.
CIN	Fluco	0.02 ± 0.01	n.c.	0.01 ± 0.00	0.13 ± 0.11	0.51 ± 0.19	n.c.	n.c.
CIN + OM	Fluco	0.04 ± 0.01	n.c.	0.02 ± 0.01	0.24 ± 0.12	0.50 ± 0.23	n.c.	n.c
CIN + CA	Fluco	0.04 ± 0.01	n.c.	0.03 ± 0.01	n.c	0.80 ± 0.20	n.c.	n.c.
CIN + OM + CA	Fluco	0.04 ± 0.01	n.c.	0.01 ± 0.00	0.30 ± 0.23	0.24 ± 0.03	n.c.	n.c.

^*a*^ The table shows the average values. C, compound; CZ-EO, *Cinnamomun zeylanicum* essential oil; AF, antifungal drug; FR2, CZ-EO fraction two; CIN, cinnamaldehayde; OM, o-methoxycinnamaldehyde; CA, cinnamyl acetate; n.c., not countable. To facilitate the understanding of the results, gray lines report the data published in the previous article (6).

Specifically, MIC values derived from FR2, CIN alone, or combinations with other components did not exhibit statistically significant variations. However, a notable distinction was observed between MIC_CZ-EO_ and MIC_CIN_ (*P* < 0.05). Data suggest that when CIN is tested at an equivalent concentration found in the CZ-EO phytocomplex (66.1%), but administered alone, it is active at lower concentrations, while the addition of other terpenes gradually leads to a decrease in CIN efficacy up to the MIC detected for CZ-EO. This behavior can be explained by the interactions established between chemicals that characterize the phytocomplex. Although the above has an impact on FIC values, it does not significantly alter the interaction with fluconazole. Notably, CZ-EO, FR2, CIN alone, or in combination with both of the two other active compounds, when mixed with fluconazole, consistently exhibited the average concentration of 0.01±0.01 % vol/vol. Similarly, the average active concentration of fluconazole, when combined with each of these natural compounds, is approximately 0.40 ± 0.33 mg/mL. In summary, the latest findings suggest that CZ-EO and CIN can be interchangeably employed to restore fluconazole effectiveness. Existing literature also supports the notion that using essential oil is a safer option compared to CIN alone, making the use of CZ-EO the preferred choice in traditional medicine. However, it is necessary to consider the known variability of phytocomplexes obtained from the seasonal distillations of an aromatic plant. This variability could hinder the development of synergistic formulations that require a rigorous balancing of active ingredients to be effective. Therefore, if future *in vivo* studies validate the efficacy and safety of CIN + fluconazole, this standardized association could be considered in the fight against resistant strains of *C. auris*.
